# Supplementary material for: Grain Yield Performance and Quality Characteristics of Waxy and Non-Waxy Winter Wheat Cultivars under High and Low-Input Farming Systems
Source: Plants (Basel). 2022 Mar 25;11(7):882. doi: 10.3390/plants11070882 (PMC9002724; doi:10.3390/plants11070882)
Supplement: Supplementary file 1 [file plants-11-00882-s001.zip › plants-1620860-supplementary.pdf]

**Supplemental Table S1.** Analysis of variance of genotypic, environmental and farming system effects on grain yield and quality characteristics of waxy and non-waxy cultivars (the table shows the sources of variation and probability for the *F* test of each factor and their interactions)

| | F-value of the source of variance | | | | | | |
|---|---|---|---|---|---|---|---|
| | Factor | | | Interaction of factors | | | |
| | Genotype | Year | Farming system | G×Y | G×F | Y×F | G×Y×F |
| **Grain yield characteristics** | | | | | | | |
| **Grain Yield** / all genotypes | 28.1** | 18.3** | 190.5** | 1.2 | 0.6 | 0.5 | 0.9 |
| waxy | 13.8** | 24.0** | 106.0** | 0.5 | 0.1 | 0.0 | 2.4 |
| non-waxy | 3.5 | 2.7 | 90.1** | 0.3 | 0.1 | 0.9 | 0.2 |
| **N use efficiency** / all genotypes | 30.1** | 22.4** | 2.0 | 1.4 | 0.2 | 3.0 | 1.0 |
| waxy | 16.4** | 29.3** | 0.5 | 1.2 | 0.4 | 1.2 | 3.5 |
| non-waxy | 3.6 | 3.7 | 1.5 | 0.5 | 0.0 | 1.9 | 0.3 |
| **Grain N yield (uptake)** / all genotypes | 41.7** | 7.9** | 233.8** | 6.0** | 2.6 | 2.7 | 1.3 |
| waxy | 18.5** | 0.6 | 119.4** | 0.3 | 0.4 | 0.0 | 2.6 |
| non-waxy | 0.8 | 15.7** | 121.2** | 3.1 | 0.1 | 3.6 | 0.1 |
| **Fertiliser N % used for grain** / all genotypes | 46.3** | 7.5* | 0.4 | 6.5** | 0.1 | 1.3 | 1.0 |
| waxy | 23.0** | 0.8 | 1.1 | 1.0 | 0.1 | 0.1 | 4.0 |
| non-waxy | 0.8 | 15.1** | 0.0 | 3.4 | 0.0 | 1.3 | 0.0 |
| **Grain chemical composition and quality characteristics** | | | | | | | |
| **Protein content** / all genotypes | 12.1** | 487.8** | 2.2 | 5.5** | 3.5* | 3.1 | 1.6 |
| waxy | 1.1 | 260.7** | 2.0 | 0.1 | 0.7 | 6.0* | 0.0 |
| non-waxy | 10.7** | 243.0** | 7.9* | 4.7* | 0.4 | 0.2 | 2.6 |
| **Phosphorus content** / all genotypes | 0.6 | 0.2 | 52.6** | 1.8 | 2.5 | 1.7 | 3.2* |
| waxy | 0.4 | 1.1 | 31.5** | 0.6 | 0.4 | 2.4 | 2.2 |
| non-waxy | 1.1 | 0.2 | 21.4** | 3.7 | 6.7** | 0.1 | 6.6** |
| **Test weight** / all genotypes | 277.4** | 1655.4** | 865.0** | 134.0** | 38.8** | 228.4** | 36.8** |
| waxy | 53.8** | 1961.5** | 223.5** | 68.3** | 1.9 | 106.2** | 0.0 |
| non-waxy | 256.8** | 276.3** | 661.2** | 107.3** | 0.4 | 123.3** | 86.8** |
| **Particle size index** / all genotypes | 365.6** | 194.0** | 19.1** | 7.2** | 0.1 | 2.8 | 2.8 |
| waxy | 17.9** | 45.0** | 11.2** | 0.0 | 0.0 | 0.1 | 0.2 |
| non-waxy | 0.6 | 169.9** | 8.0* | 2.4 | 0.0 | 7.4* | 3.7 |
| **Sedimentation** / all genotypes | 437.3** | 164.7** | 11.9** | 21.2** | 10.1** | 54.8** | 4.1* |
| waxy | 0.5 | 47.5** | 4.1 | 0.6 | 0.2 | 15.7** | 0.6 |
| non-waxy | 6.1** | 117.4** | 26.9** | 19.4** | 0.0 | 39.2** | 0.2 |
| **Falling number** / all genotypes | 16444.2** | 276.4** | 22.7** | 93.9** | 9.6** | 121.3** | 41.6** |
| waxy | 160.2** | 0.2 | 8.2* | 4.2 | 1.5 | 1.5 | 0.2 |
| non-waxy | 2.7 | 276.6** | 24.5** | 3.5 | 1.4 | 123.4** | 0.1 |
| **Wet gluten content** / all genotypes | 1.9 | 101.1** | 4.8* | 12.9** | 3.8* | 0.2 | 0.8 |
| waxy | 6.09* | 35.3** | 0.8 | 2.1 | 0.0 | 1.7 | 0.4 |
| non-waxy | 0.5 | 67.1** | 9.1** | 13.1** | 1.2 | 0.0 | 1.1 |
| **Gluten index** / all genotypes | 38.3** | 42.3** | 1.1 | 11.1** | 1.0 | 2.9 | 1.0 |
| waxy | 21.4** | 44.5** | 0.1 | 5.4* | 0.1 | 0.3 | 0.3 |
| non-waxy | 2.6 | 2.5 | 6.13* | 4.0 | 0.5 | 5.6* | 3.6 |
| **Gluten water absorption** / all genotypes | 13.7** | 72.2** | 3.8 | 9.0** | 2.0 | 18.0** | 4.0* |
| waxy | 7.4* | 50.7** | 3.4 | 0.1 | 2.0 | 16.2** | 1.0 |
| non-waxy | 5.3** | 25.0** | 0.4 | 17.7** | 1.7 | 1.9 | 0.2 |
| **Flour and dough quality characteristics** | | | | | | | |
| **Flour yield** / all genotypes | 342.7** | 77.5** | 0.4 | 2.7 | 8.0** | 42.1** | 1.5 |
| waxy | 56.6** | 40.2** | 4.2 | 2.1 | 8.7** | 24.9** | 0.0 |
| non-waxy | 0.0 | 39.2** | 3.4 | 4.2 | 7.6* | 17.5** | 2.8 |

| | | | | | | | |
|---|---|---|---|---|---|---|---|
| **Flour water absorption (BF)** / all genotypes | 2139.7** | 39.2** | 0.1 | 1.9 | 1.7 | 5.1* | 0.6 |
| waxy | 315.2** | 7.5* | 1.5 | 0.6 | 1.3 | 1.2 | 0.6 |
| non-waxy | 2.8 | 56.3** | 2.2 | 4.3 | 0.0 | 6.4* | 1.8 |
| **Dough development time (BF)** / all genotypes | 99.3** | 14.3** | 0.2 | 8.9** | 4.7* | 19.8** | 8.0** |
| waxy | 108.4** | 0.1 | 0.4 | 0.5 | 7.0* | 23.5** | 1.4 |
| non-waxy | 20.4** | 138.6** | 0.2 | 20.8** | 5.5* | 0.1 | 0.9 |
| **Dough stability time (BF)** / all genotypes | 3.5* | 1.7 | 1.5 | 1.0 | 4.4* | 14.5** | 2.1 |
| waxy | 10.0** | 0.6 | 0.1 | 2.3 | 8.8** | 3.2 | 0.0 |
| non-waxy | 0.0 | 1.2 | 2.1 | 0.7 | 4.2 | 12.2** | 3.7 |
| **Degree of softening10 (BF)** / all genotypes | 24,3** | 4,8* | 0,0 | 1,6 | 3,3* | 12,8** | 1,0 |
| waxy | 40.4** | 0.0 | 0.7 | 0.1 | 2.3 | 2.6 | 1.0 |
| non-waxy | 3.4 | 22.1** | 2.4 | 0.3 | 10.1** | 22.4** | 2.2 |
| **Degree of softening12 (BF)** / all genotypes | 7,2** | 13,3** | 10,6** | 11,8** | 8,0** | 66,1** | 1,6 |
| waxy | 8.5** | 13.2** | 4.8* | 17.5** | 11.6** | 22.3** | 2.9 |
| non-waxy | 2.2 | 0.9 | 8.1* | 0.5 | 12.3** | 72.0** | 0.0 |
| **BF quality number (BF)** / all genotypes | 18,2** | 9,2** | 3,2 | 4,1* | 7,6* | 28,5** | 0,1 |
| waxy | 43.1** | 0.0 | 0.1 | 0.4 | 7.0* | 13.5** | 0.2 |
| non-waxy | 3.0 | 20.1** | 5.4* | 1.6 | 14.0** | 15.0** | 0.2 |
| **Starch content and quality characteristics** | | | | | | | |
| **Starch content** / all genotypes | 19,2** | 84,4** | 19,9** | 0,6 | 1,6 | 2,3 | 0,9 |
| waxy | 2.9 | 57.7** | 13.4** | 1.0 | 4.1 | 2.2 | 2.7 |
| non-waxy | 5.2* | 31.2** | 7.5* | 0.4 | 1.1 | 0.5 | 0.0 |
| **A-type starch** / all genotypes | 5,4** | 265,5** | 7,7** | 3,8* | 1,0 | 5,0* | 2,3 |
| waxy | 8.1* | 407.3** | 21.7** | 6.3* | 2.4 | 24.6** | 4.4 |
| non-waxy | 0.2 | 66.8** | 0.6 | 3.9 | 0.1 | 0.0 | 0.3 |
| **Amylose content** / all genotypes | 5539,3** | 29,3** | 1,1 | 5,4** | 2,0 | 0,7 | 0,7 |
| waxy | 3.4 | 1.6 | 0.9 | 0.6 | 0.1 | 0.0 | 0.2 |
| non-waxy | 0.9 | 37.8** | 5.1* | 1.5 | 0.5 | 1.6 | 0.6 |
| **Peak viscosity (RVA)** / all genotypes | 78,2** | 53,8** | 1,7 | 0,7 | 0,9 | 1,8 | 0,3 |
| waxy | 1.5 | 20.7** | 2.0 | 0.1 | 0.4 | 1.2 | 0.1 |
| non-waxy | 18.9** | 84.9** | 0.0 | 2.2 | 0.7 | 0.9 | 1.1 |
| **Trough (RVA)** / all genotypes | 117,5** | 343,79** | 1,2 | 9,0** | 1,0 | 1,0 | 0,4 |
| waxy | 7.0** | 162.9** | 4.8** | 11.6** | 0.2 | 1.8 | 0.4 |
| non-waxy | 1.5 | 187.6** | 0.1 | 3.1 | 0.6 | 0.1 | 0.4 |
| **Breakdown (RVA)** / all genotypes | 272,0** | 3,3 | 0,9 | 5,0** | 0,5 | 1,2 | 0,1 |
| waxy | 0.3 | 1.0 | 0.9 | 0.6 | 0.4 | 0.7 | 0.0 |
| non-waxy | 13.2** | 77.5** | 0.0 | 0.6 | 0.0 | 0.5 | 0.1 |
| **Final Viscosity (RVA)** / all genotypes | 463,8** | 406,7** | 2,4 | 5,8** | 0,4 | 3,3 | 0,5 |
| waxy | 2.8 | 201.7** | 3.9 | 12.0** | 0.0 | 3.9 | 0.4 |
| non-waxy | 0.2 | 205.8** | 0.1 | 4.9* | 0.1 | 0.4 | 0.5 |
| **Setback (RVA)** / all genotypes | 464,2** | 58,2** | 1,2 | 6,2** | 0,3 | 2,7 | 0,2 |
| waxy | 0.2 | 55.6** | 0.3 | 2.3 | 0.1 | 2.3 | 0.1 |
| non-waxy | 1.9 | 8.7** | 1.1 | 1.3 | 1.1 | 0.6 | 0.1 |
| **Peak Time (RVA)** / all genotypes | 2945,4** | 81,1** | 4,5* | 25,4** | 1,0 | 0,0 | 2,8 |
| waxy | 1.0 | 0.2 | 0.5 | 0.4 | 0.9 | 1.1 | 0.4 |
| non-waxy | 0.0 | 105.7** | 4.2 | 1.5 | 0.3 | 0.2 | 5.0* |
| **Viscosity temperature (RVA)** / all genotypes | 138,7** | 1,6 | 1,6 | 0,7 | 0,6 | 0,9 | 2,4 |
| waxy | 0.3 | 1.2 | 0.2 | 0.2 | 0.0 | 0.3 | 1.5 |
| non-waxy | 1.2 | 1.2 | 1.4 | 0.6 | 0.3 | 1.2 | 3.0 |

G – genotype, Y– year, F – farming system, BF – Brabender farinograph; RVA – rapid visco analyser; * and ** significant at P<0.05 and 0.01, respectively.
